# Plant Metabolites Affect *Fusarium proliferatum* Metabolism and In Vitro Fumonisin Biosynthesis

**DOI:** 10.3390/ijms24033002

**Published:** 2023-02-03

**Authors:** Justyna Lalak-Kańczugowska, Natalia Witaszak, Agnieszka Waśkiewicz, Jan Bocianowski, Łukasz Stępień

**Affiliations:** 1 Institute of Bioorganic Chemistry, Polish Academy of Sciences, 61-704 Poznań, Poland; 2 Institute of Plant Genetics, Polish Academy of Sciences, 60-479 Poznań, Poland; 3 Department of Chemistry, Poznań University of Life Sciences, 60-625 Poznań, Poland; 4 Department of Mathematical and Statistical Methods, Poznań University of Life Sciences, 60-637 Poznań, Poland

**Keywords:** *Fusarium*, gene expression, metabolites, mycotoxins, plant-pathogen interaction, qPCR

## Abstract

*Fusarium proliferatum* is a common hemi-biotrophic pathogen that infect a wide range of host plants, often leading to substantial crop loss and yield reduction. *F. proliferatum* synthesizes various mycotoxins, and fumonisins B are the most prevalent. They act as virulence factors and specific effectors that elicit host resistance. The effects of selected plant metabolites on the metabolism of the *F. proliferatum* strain were analyzed in this study. Quercetin-3-glucoside (Q-3-Glc) and kaempferol-3-rutinoside (K-3-Rut) induced the pathogen’s growth, while DIMBOA, isorhamnetin-3-*O*-rutinoside (Iso-3-Rut), ferulic acid (FA), protodioscin, and neochlorogenic acid (NClA) inhibited fungal growth. The expression of seven *F. proliferatum* genes related to primary metabolism and four *FUM* genes was measured using RT-qPCR upon plant metabolite addition to liquid cultures. The expression of *CPR6* and *SSC1* genes was induced 24 h after the addition of chlorogenic acid (ClA), while DIMBOA and protodioscin reduced their expression. The transcription of *FUM1* on the third day of the experiment was increased by all metabolites except for Q-3-Glc when compared to the control culture. The expression of *FUM6* was induced by protodioscin, K-3-Rut, and ClA, while FA and DIMBOA inhibited its expression. *FUM19* was induced by all metabolites except FA. The highest concentration of fumonisin B_1_ (FB_1_) in control culture was 6.21 µg/mL. Protodioscin did not affect the FB content, while DIMBOA delayed their synthesis/secretion. Flavonoids and phenolic acids displayed similar effects. The results suggest that sole metabolites can have lower impacts on pathogen metabolism and mycotoxin synthesis than when combined with other compounds present in plant extracts. These synergistic effects require additional studies to reveal the mechanisms behind them.

## 1. Introduction

*Fusarium proliferatum* is a common hemi-biotrophic pathogen that infects a wide range of host plants including cereals, legumes, and vegetable and fruit crops and can be persistent for many years [1]. When a pathogen encounters a susceptible plant under favorable conditions, the infection often leads to substantial crop loss and yield reduction [2,3,4]. *F. proliferatum* is mainly transferred and spread by seeds and crop residues. Infection at early seedling stages kills the plant and at later stages results in poor yield [5]. During the infection, *F. proliferatum* synthesizes secondary metabolites called mycotoxins that weaken the host defense mechanisms. *F. proliferatum* synthesizes a plethora of mycotoxins, such as beauvericin, fusaproliferin, moniliformin, fusaric acid, and fusarins, but fumonisin B toxins are the most prevalent among them [6]. Some reports indicate that fumonisin B_1_ is a virulence factor causing FB_1_-induced cell death, mediated by ROS activation, phytoalexin accumulation, and PR gene overexpression [7,8]. They also act as specific effectors that elicit host systemic acquired resistance (SAR), which mainly involves the activation of the salicylic acid (SA) signaling pathway [9]. Plant metabolites involved in disease resistance are flavonoids, phenylpropanoids, and polyamines. Phenolic compounds including coumaric acid, coumarin, quercetin, etc., are the largest group of plant antioxidant phytochemicals from which many products are synthesized [10].

Several plant secondary metabolites were found to have growth inhibitory effects on many plant-pathogenic fungi and bacteria. These include polyamines, flavonoids, and phytoalexins [11,12,13,14,15,16]. In contrast, some concentrations of plant phenolic compounds can stimulate the growth of *Fusarium* fungi [17]. Flavonoids help plants to fight the pathogen through various mechanisms and along with polyamines suppress reactive oxygen species (ROS) produced during the infection [18,19,20]. Additionally, flavonoids have an inhibitory effect on trichothecene synthesis by inhibiting cytochrome P450 monooxygenase, which converts trichodiene to oxygenated trichothecenes [21]. Phenolic compounds such as ferulic acid, p-hydroxybenzoic acid, and vanillic acid are proven to reduce the aurofusarin and zearalenone toxin levels in *Fusarium* species [17]. Phenylpropanoid antifungal properties result in higher resistance levels of plants with high phenylpropanoid content than those with lower ones [22,23,24].

Plant–pathogen interactions comprise a complex process involving actions and reactions. To understand it fully, it is often necessary to examine detailed changes in both organisms during the infection process. Our previous studies on *F. proliferatum* indicated that an asparagus extract affected *FUM* gene expression and changed the biosynthesis of fumonisins [1,25,26]. Interestingly, the original research revealed that host extracts are able to exert generally similar effects on the metabolism of *F. proliferatum* strains regardless of the origin [1]. Further proteomic analyses showed the proteins induced by the asparagus extract [27]. However, it was not sufficient to ultimately explain the metabolic mechanisms of *F. proliferatum*. Therefore, the aim of this study was to investigate the effect of plant bioactive compounds on fumonisins production, fungal growth, and primary and secondary metabolism of the *Fusarium proliferatum* strain. Seven genes related to primary metabolism were chosen based on our previous research on the *Fusarium proliferatum* response to biotic stress [27]: *CPR6* (encoding cytosolic cyclophilin), *FeSOD* (superoxide dismutase), *HSP70*, *HSP88* and *SSC1* (heat shock proteins), *SpD* (saccharopin dehydrogenase), and *UOR* (NADH-Q oxidoreductase). Additionally, four genes from the *FUM* biosynthetic gene cluster (*FUM1*, *FUM6*, *FUM8* and *FUM19*) were included.

## 2. Results

### 2.1. Effect of Plant Metabolites on Fungal Growth

**Figure 1:**
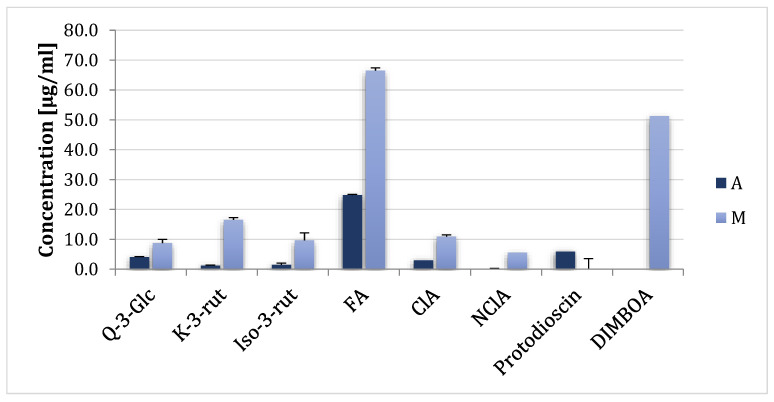
Concentrations of plant metabolites used in the study in water extracts from asparagus spears and maize ears. A—asparagus, M—maize. Values are means of three repetitions ± standard deviation.

Eight plant metabolites (Table 1) used in the study were selected on the basis of previous research, as well as based on literature data [28,29,30]. Originally, the metabolites were quantified in asparagus and maize extracts (Figure 1).

**Figure 2:**
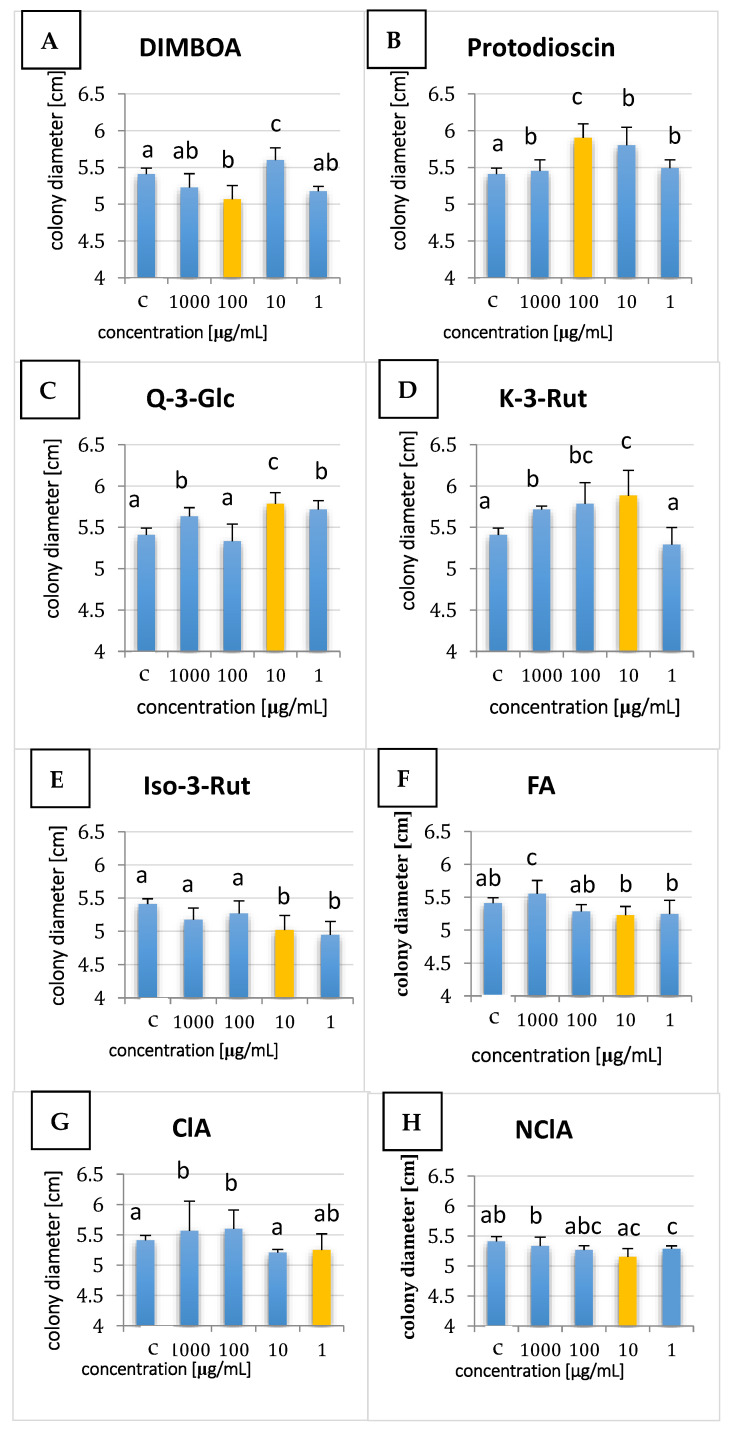
Colony diameters of the *F. proliferatum* KF 3360 strain under three concentrations of eight plant metabolites (details in Table 1) after six days of cultivation. Panels (**A**—**H**) show results for individual metabolites studied. C stands for controls, values are means of three repetitions ± standard deviation (HSD Tukey test, *p* < 0.05). Yellow bars represent the concentration used for liquid cultures and gene expression analyses.

Three concentrations were evaluated compared to the control for seven days, and results for the sixth day of culturing are shown in Figure 2. Quercetin-3-glucoside (Q-3-Glc) and kaempferol-3-rutinoside (K-3-Rut) induced growth of KF 3360, but the majority of the metabolites inhibited fungal growth: DIMBOA, isorhamnetin-3-*O*-rutinoside (Iso-3-Rut), ferulic acid (FA), protodioscin, and neochlorogenic acid (NClA). For chlorogenic acid (ClA), the reaction of the strain was concentration-dependent, and only higher amounts stimulated fungal growth (Figure 2).

### 2.2. Expression Changes of Primary Metabolic Genes in the Presence of Plant Metabolites

**Figure 3:**
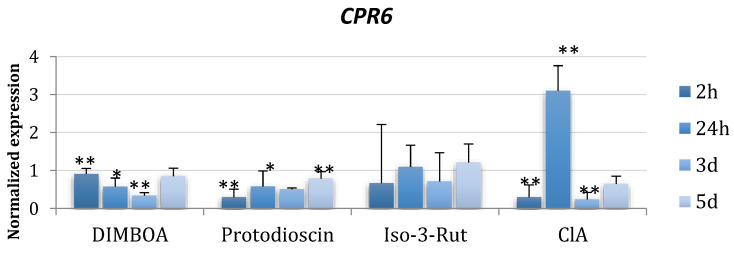

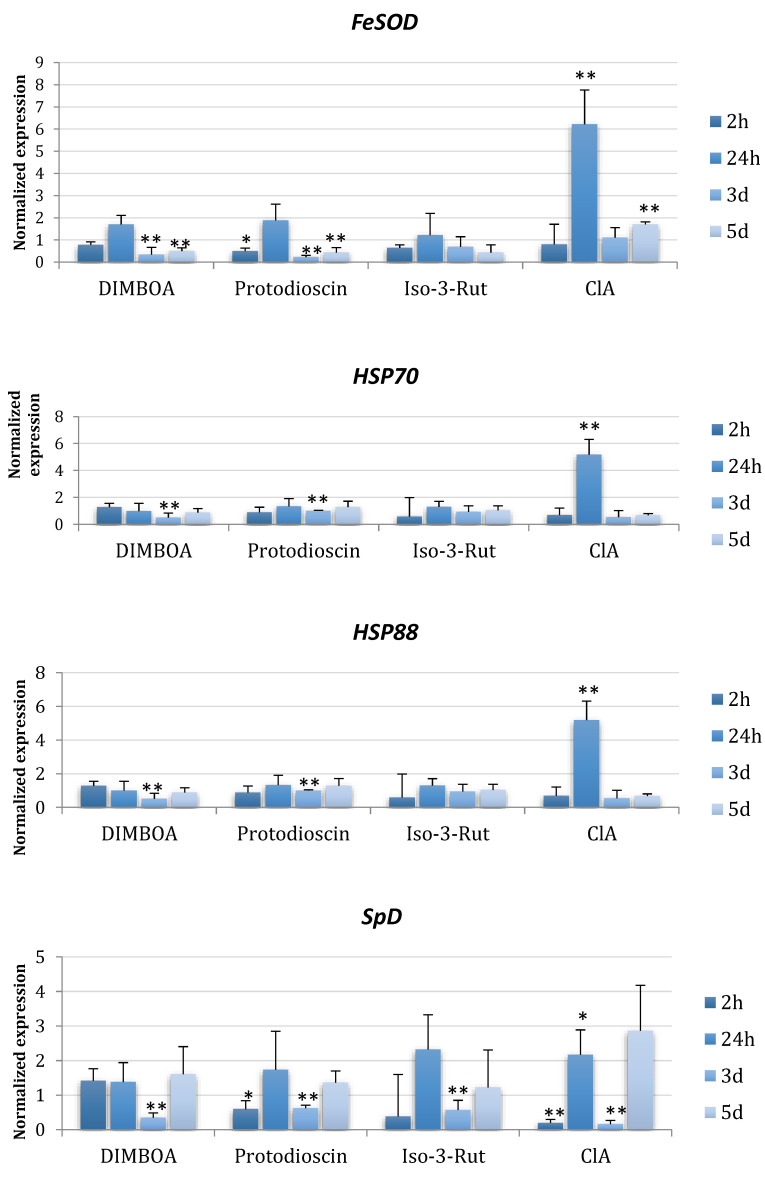

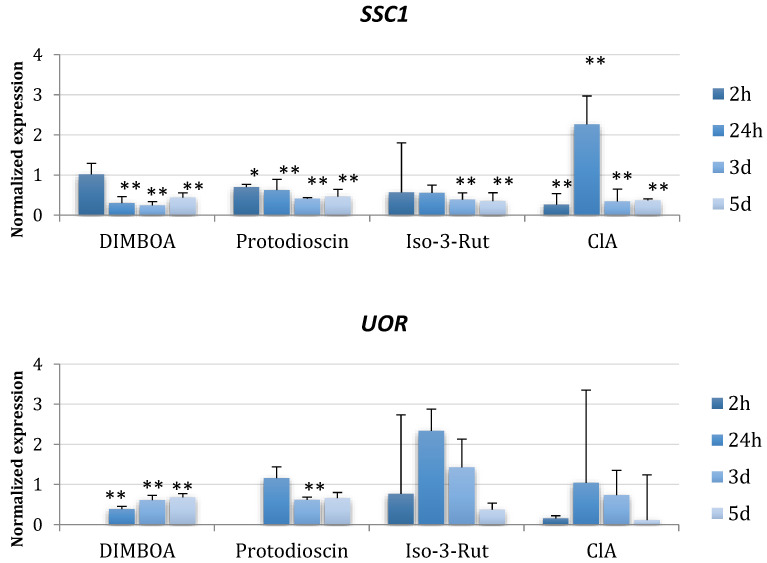
Changes in expression of the chosen primary metabolism-linked genes induced by the plant metabolites in liquid *Fusarium proliferatum* KF 3360 cultures after 2, 24, 72, and 120 h after metabolite addition. Average values of three replicates are shown. Differences statistically significant at * *p* < 0.05, ** *p* < 0.01, and standard deviations are shown.

Expression levels were analyzed for seven *F. proliferatum* genes related to the primary metabolism (Table 2). Genes were chosen based on previous works [25]. As plant metabolites, protodioscin, isorhamnetin-3-*O*-rutoside, DIMBOA, and chlorogenic acid were shown here based on fumonisin biosynthesis and *FUM* gene expression results (see below). For the results obtained using the remaining metabolites, please see Supplementary Tables S1 and S2. For the majority of genes analyzed, the expression levels were lower than the in the control cultures, regardless of the time point (2 h and 1, 3, and 5 days after metabolites were added) (Figure 3, Table S1).

*CPR6* and *SSC1* genes showed induced expression 24 h after addition of ClA, while DIMBOA and protodioscin reduced their expression (Figure 3). Expression changes induced by chlorogenic acid for *FeSOD, HSP88, SpD*, and *UOR* were more evident than for other metabolites. The *Hsp88* gene was the least prone to the action of the compounds. Only a slight decrease was observed at day 3 after DIMBOA addition and an increase at day 1 for ClA. Iso-3-Rut did not affect the expression of *CPR6, FeSOD*, and *HSP88* genes substantially. On the other hand, *UOR* gene expression was lowered by DIMBOA and protodioscin and induced by Iso-3-Rut at day 1 and 3.

### 2.3. Expression of FUM Genes Governing Fumonisin Biosynthesis

**Figure 4:**
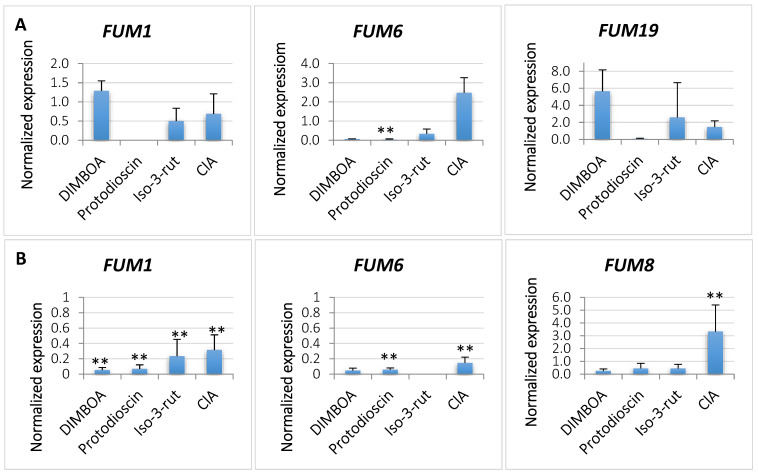
Changes in expression of expression of four *FUM* genes induced by the plant metabolites in liquid *Fusarium proliferatum* KF 3360 cultures after 3 (**A**) and 5 days (**B**) after metabolite addition when compared to the control cultures. Average values of three replicates are shown. Differences statistically significant at ** *p* < 0.01, and standard deviations are shown.

Samples collected on day 3 and 5 of culturing were subjected to the RT-qPCR analysis of four *FUM* genes. Transcription of *FUM1* on day 3 was increased by all metabolites except Q-3-Glc when compared to the control culture (Figure 4, Supplementary Table S2). For *FUM6* the situation was different. It was induced by protodioscin, K-3-Rut, and ClA, while FA and DIMBOA inhibited its expression. *FUM19* was induced by all metabolites except FA but only on day 3, because on day 5 the expression was not observed. In general, all *FUM* genes displayed lower expression on day 5 compared to the controls, except for *FUM8* (Figure 4, Supplementary Table S2).

### 2.4. Effect of Plant Metabolites on Fumonisins Biosynthesis

**Figure 5:**
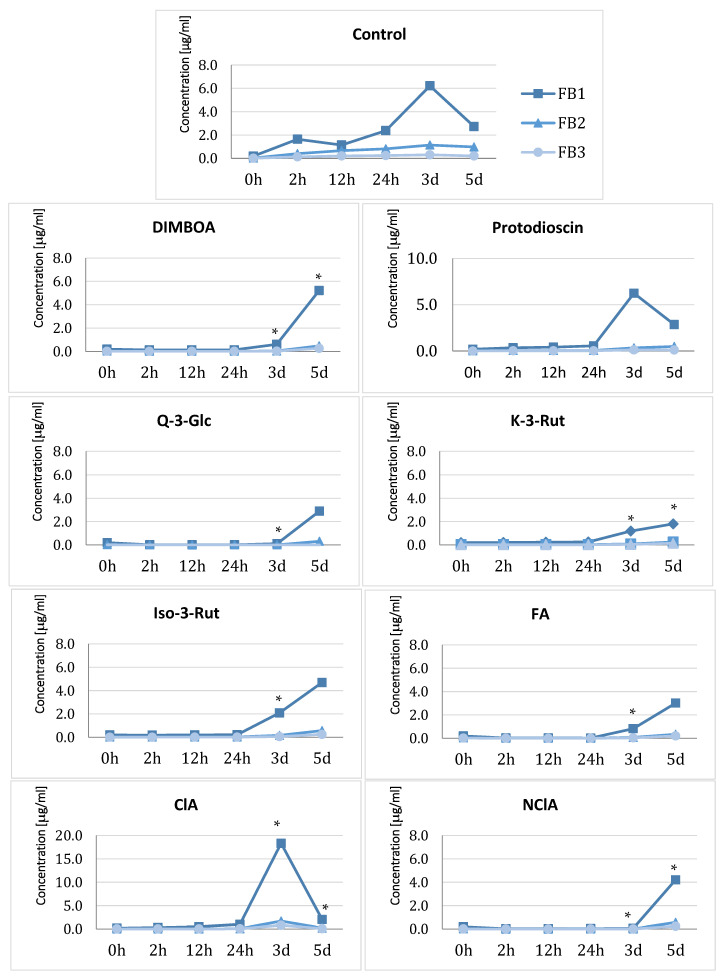
Changes in fumonisins concentrations in *F. proliferatum* KF 3360 cultures after the addition of plant metabolites: ■—FB_1_, ▲—FB_2_, ●—FB_3_. * differences statistically significant compared to control at *p* < 0.05. All analyses were done in triplicate. Detailed information along with SDs are included in Supplementary Table S3.

Fumonisin contents in control culture media increased already 12 h after starting the experiment, and at day 3 the highest concentration of FB_1_ observed was 6.21 µg/mL (Figure 5). The addition of plant metabolites to the cultures usually gradually increased the content of FBs in the media. In the cases of DIMBOA, kaempferol-3-rutinoside, isorhamnetin-3-*O*-rutoside, and ferulic acid, the increase was observed after 24 h of metabolite addition, and for quercetin-3-glucoside and neochlorogenic acid after 3 days. The highest mycotoxin content for the majority of the metabolites was in the range of 4–6 µg/mL and was measured in samples collected on the third day of cultivation (Figure 5). The only exception was chlorogenic acid, which induced significantly fumonisin synthesis, however only at the third day of culture when the medium contained almost 20 µg/mL of FB_1_.

## 3. Discussion

*Fusarium proliferatum* is capable of inhabiting a broad spectrum of host plant species, including maize, garlic, asparagus, pineapple, date palm, and onion [1,3], and this list is growing constantly due to climate change. Consequently, plants also evolve mechanisms to identify and respond to new potential pathogens [31,32]. While extensive research on environmentally friendly disease control microorganisms is well-established, recently, plant-derived extracts and bioactive compounds have gained substantial attention in plant protection studies [14,33,34]. In our previous studies we have shown the characteristics of asparagus extract and its fraction concerning the interaction with *F. proliferatum* strains and their metabolism [25,26]. Here, based on the quantitative analysis of asparagus and maize extracts, individual metabolites were chosen to analyze their influence on fungal growth, metabolism, and fumonisin synthesis. This process is governed by the *FUM* gene cluster, which was already well-recognized and described for at least three *Fusarium* species and also other fungal species [35,36,37,38,39]. The function of individual genes were established and confirmed using knock-out mutants of *Fusarium verticillioides*, which is being considered as a model producer for this group of mycotoxins [35].

Polyamines and phenolic acids (including flavonoids) are known for their antioxidant properties, though members of these two groups vary in anti-oxidative potential due to the structure and number of moieties attached. Glycosylation of flavonoids increases their solubility in water, which facilitates the transport but reduces chemical reactivity at the same time [40]. Double-glycosylated Iso-3-Rut and K-3-Rut show less anti-oxidant ability than single-glycosylated Q-3-Glc.

It was proven that phenolic compounds such as *p*-hydroxybenzoic acid, rutin, quercetin, and vanillic acid change the metabolism of *Fusarium* species in a dose-dependent manner, and the fungal response is not species-specific [14]. This, however, was not fully confirmed in our study, as amounts of fungal biomass did not differ significantly in cultures with various concentrations of plant metabolites (Figure 2).

Our previous studies showed that *F. proliferatum* metabolism changes upon the addition of host plant extracts [1,25,27]. However, individual metabolites present in those extracts were not tested until now. The regulation of primary metabolism is a complex issue; however, it could be proven that *F. proliferatum* HSP70 and SOD proteins (encoded by the genes studied also in the present study) are expressed differentially in relation to the pH and phenolic compound content in the medium [41].

Fumonisin biosynthesis was differentially affected by the metabolites tested (Figure 5). Although some researchers found a good correlation between *FUM* gene expression and fumonisin content [42], we were not able to confirm it, possibly due to the methodology used, because the expression of the genes was measured for mycelia, while fumonisins were only quantified in the culture media. It is known that substrate composition is related with the amounts of fumonisins synthesized [43,44,45,46], and it is critical for the comparison of the results of individual studies [47]. Moreover, low concentrations of FBs in the media and simultaneous higher expression of *FUM* genes may be explained by the inhibition of mycotoxin excretion or their accumulation in intracellular compartments, giving the protection against oxidative stress [47,48]. A similar effect was recorded for *F. verticillioides*, where plant extract fractions inhibited FB biosynthesis without impairing its growth [49]. The increased accumulation of FBs in mycelia than in media was already observed in our previous studies [25].

In the present study, protodioscin did not affect the FB content compared to the control culture, while DIMBOA delayed their synthesis/secretion. Flavonoids and phenolic acids displayed similar effects. This is in agreement with the results of Ferrochio et al. [16], who found FBs and growth inhibition by ferulic acid at concentrations higher than 20 mM. In addition, in *F. sporotrichioides* and *F. langsethiae*, ferulic acid influenced the biosynthesis of T-2 and HT-2 toxins and the expression of the *TRI* gene cluster responsible for trichothecene biosynthesis [50]. On the other hand, there are reports stating that low concentrations of ferulic, cinnamic, and caffeic acids inhibit fumonisins but not growth of the mycelium [51]. These discrepancies are perhaps due to variation of culture media and metabolite concentrations. It has been suggested that resistance to pathogens may be associated with a higher content of phenylpropanoids [19,20,52,53]. Similarly, a deficit of saponins can lead to lower plant resistance [54]. Phenolic compounds (such as ferulic acid and *p*-coumaric acid) protect plant cell wall components from being degraded by a pathogen’s hydrolytic enzymes [55], which limits the fungal spread [17,21,56]. Future studies should evaluate the possible synergistic effects of individual metabolites, as well as the action of their derivatives, as a possible role of some of the tested metabolites (such as chlorogenic acid and quercetin) in plant resistance to fungal infection was already suggested [57].

## 4. Materials and Methods

### 4.1. Fungal Strain and Culture Conditions

*Fusarium proliferatum* strain KF3360 was originally isolated from asparagus spear in 2009 in Poland, and it was stored in the KF collection of pathogenic fungi at the Institute of Plant Genetics, Polish Academy of Sciences, Poznań, Poland. The strain was identified using molecular techniques during previous studies [1] and maintained on potato dextrose agar (PDA, Oxoid, Basingstoke, UK) medium at 25 °C for 7 days.

### 4.2. Plant Extract Preparation

Water extracts were prepared from white asparagus spears and young maize cobs according to the procedure described earlier [1]. Fresh white asparagus spears and young maize cobs without any disease symptoms were frozen overnight at −80 °C and homogenized the next day in a blender. The obtained mass was centrifuged at 6000× *g* for 15 min at 4 °C. Extracts were filtered through 0.20 μm membrane (Chromafil PET20/15 MS, Macherey-Nagel, Dueren, Germany) and stored at –20 °C.

### 4.3. Growth Rate Measurement

*Fusarium proliferatum* KF 3360 strain cultures on solid PDA medium were used to evaluate the efficiency of plant metabolites in reducing the fungal growth in vitro. Four arbitrary concentrations of plant metabolite standards, namely, quercetin-3-glucoside, kaempferol-3-rutinoside, isorhamnetin-3-*O*-rutinoside, ferulic acid, chlorogenic acid, neochlorogenic acid, protodioscin, DIMBOA (all supplied by Sigma Aldrich, Taufkirchen, Germany) were tested (1000, 100, 10, and 1 μg/mL). Sterile 90 mm Petri plates contained 15 mL of PDA medium and were inoculated with a single PDA plug (1 cm^2^) of mycelium. The solutions of plant metabolites were then applied onto the plug and then incubated for seven days at room temperature with a 12 h photoperiod. The experiment was conducted in triplicate, and colony diameter was measured daily. For protodioscin and DIMBOA, 100 μg/mL concentration was chosen for liquid cultures and 1 μg/mL for chlorogenic acid, while 10 μg/mL was used for the remaining compounds.

### 4.4. Liquid Cultures with Plant Metabolite Standards

All cultures were conveyed in vitro in 100 mL flasks containing 45 mL of a fumonisin-inducing liquid medium (25 °C without shaking at 12 h photoperiod) [1]. The medium contained yeast extract 1 g/L, malt extract 0.5 g/L, mycological peptone 1 g/L, KH_2_PO_4_ 1 g/L, MgSO_4_ × 7 H_2_O 0.3 g/L, KCl 0.3 g/L, ZnSO_4_ × 7 H_2_O 0.05 g/L, CuSO_4_ × 5 H_2_O 0.01 g/L, and D-fructose 20 g/L. About 4 cm^2^ of mycelium harvested of the PDA plates was used for inoculation. At the 5th day of cultivation, the culture was supplemented with 30 μL of plant metabolite standard solutions at the concentration selected based on the growth measurement experiment. The negative control was supplemented with equal amounts of sterile water. Media samples were collected in the following time points: 0 h, 2 h, 24 h, 72 h, and 120 h after metabolites were added to the cultures and subjected to the quantification of fumonisins. All cultures were done in triplicate.

### 4.5. Mass Spectrometry Analysis

An Acquity UPLC system (Waters Corporation, Milford, MA, USA) combined with a Q-Exactive high-resolution mass spectrometer (Thermo Fisher, Waltham, MA, USA) with an Orbitrap mass analyzer was used. Samples (5 μL) were injected onto an ACQUITY UPLC BEH Shield RP18 column (150 × 2.1 mm, particle size 1.7 μm) (Waters, Manchester, MA, USA), with a flow rate of 0.35 μL min^−1^ at 50 °C. Mobile phases contained 0.1% (*v*/*v*) formic acid in water (A) (LC-MS grade, Merck, Darmstadt, Germany) and acetonitrile (B) (LC–MS grade, Merck). A multi-step linear gradient was as follows: 5% B—1.5 min, 80% B—10.5 min, 98% B—11.5 min, 5% B—13 min.

Mass spectrometry analysis was performed using heated electrospray ionization (H-ESI) in positive and negative modes. A 3.5 kV and 2.5 kV ion spray voltage was applied for positive and negative ionization, respectively. Ion source temperature was 320 °C. Data were acquired in Full MS/data-dependent MS2 mode in the 100–1500 *m/z* range. The resolution of Full MS was 70,000 and of ddMS2 17,500. Normalized collision energy in the ddMS2 experiment was set to 30%. Xcalibur software (ThermoFisher Scientific, Waltham, MA, USA) was used for system operation, data acquisition, and data analysis [58].

### 4.6. Plant Metabolite Identification in Asparagus and Maize Extracts

Metabolite identification was done based on accurate mass and ion fragmentation patterns in Xcalibur Qual Browser (ThermoFisher Scientific, Waltham, MA, USA) and statistically analyzed using Perseus (version 1.6.1.3.) [59]. Eight plant metabolites were selected for the present study based on this identification (Table 1).

### 4.7. Gene Expression Analyses

RT-qPCR was used to analyze the expression of seven primary metabolism genes and four genes from the *FUM* gene cluster responsible for fumonisin biosynthesis in *F. proliferatum* after the addition of plant metabolites (Table 2). Samples of mycelia collected after 0 h, 2 h, 24 h, 72 h, and 120 h of culturing were frozen in liquid nitrogen and lyophilized. Then, after homogenization in a ball mill (Retsch-Qiagen, Manchester, UK), total RNA was extracted using a Universal RNA Purification Kit (EURx, Gdańsk, Poland), followed by treatment with RNase-free DNase (Qiagen, Manchester, UK). The RNA concentration was measured using a NanoDrop ND-1000 (Thermo Fisher Scientific, Waltham, MA, USA) and RNA quality and integrity were evaluated on 1% agarose gel (100 V/20 min). Then, 1 μg of total RNA was reverse-transcribed using a High Capacity cDNA Reverse Transcription Kit (Applied Biosystems, Foster City, CA, USA). Reactions were incubated at 25 °C for 10 min, followed by 37 °C for 120 min and 85 °C for 5 min in a C-1000 thermal cycler (Bio-Rad, Hercules, CA, USA). Primers were designed using PrimerQuest (Integrated DNA Technologies) and are listed in Table 2. Originally, three reference genes were tested: *TEF*-1α, *Tub2*, and *H3*. However, only the *H3* gene showed stable expression in all conditions. All primers were validated during a preliminary experiment.

The resulting cDNA was used as a template for RT-qPCR (SsoAdvanced Universal SYBR Green Supermix) using the Bio-Rad^TM^ CFX96^TM^ system (Bio-Rad, Hercules, CA, USA) with H3 histone as a reference gene used for normalizing the differences in mRNA quantities. Three biological and two technical replicates of each sample were included in each assay. Target sequences were amplified in a 5 μL volume containing 2.5 μL of SsoAdvanced universal SYBR Green supermix, an appropriate amount of each primer, and 2 μL of the cDNA template. The PCR cycling conditions were initial denaturation at 95 °C for 30 s followed by 40 cycles of denaturation at 95 °C for 10 s and annealing at 62–68 °C for 30 s. The melting curve analysis from 65–95 °C with a 0.5 °C increment (5 s per step) confirmed primer pairs specificity. The results were analyzed using CFX Maestro 1.1 software (Bio-Rad, Hercules, CA, USA).

### 4.8. Fumonisin Quantification

Ultrapure fumonisin standards (FB_1–3_, 50 μg/mL in acetonitrile: water, 1:1), LC/MS-grade organic solvents, and reagents were supplied by Sigma-Aldrich (Steinheim, Germany). Water was purified using a Milli-Q system (Millipore, Bedford, MA, USA). The analytical system consisted of the Aquity UPLC chromatograph (Waters, Manchester, MA, USA), coupled with an electrospray ionization triple quadrupole mass spectrometer (TQD) (Waters, Manchester, MA, USA). A Waters ACQUITY UPLC HSS T3 (100 × 2.1 mm/ID, with a particle size of 1.8 μm) was used for chromatographic separation with a flow rate of 0.35 mL/min (room temperature). Mobile phases were methanol (A) and water (B), both containing 0.1% formic acid and additionally 2 mM ammonium formate. The following gradient was used: from 1% to 95% A in 10 min, then 95% A for 2 min, and return to initial conditions for 2 min. The injection volume was 3 μL. The mass spectrometer was set to positive ionization mode. The ion source/desolvation temperatures were 150/350 °C, respectively. Nebulizing gas (nitrogen) flow rate was 750 L/min, cone flow rate was 20 L/min. Argon was used as the collision gas, with the energy of 14–22 eV. The metabolites were identified by comparing the retention times, and *m/z* values were obtained by MS and MS2 with the mass spectra (722.4/352.4, 706.4/336.4, and 706.4/170.4 for FB_1_, FB_2_, and FB_3_, respectively). LOD was 0.1 ng/μL. All samples were analyzed in triplicate. For data processing, EmpowerTM 1 software was used (Waters, Manchester, UK).

### 4.9. Statistical Analyses

One-way analysis of variance (ANOVA) followed by the Tukey test (HSD) (5% level of significance) was used to evaluate differences of FB_1_, FB_2_, and FB_3_ synthesis between control and cultures with plant metabolites added during the incubation. The results were analyzed using STATISTICA 13.1 software. Mean values (*n* = 3) and standard errors were calculated. Target gene expression (linked to primary and secondary metabolism) was determined using the 2^−∆∆Ct^ method [60] and normalized against reference genes (H3 histone). All data were analyzed using CFX Maestro 1.1 software (Bio-Rad, Hercules, CA, USA). The differences between samples were evaluated using ANOVA (5% level of significance). The expression was transformed to ln(x), and baseline correction and threshold were set automatically in the CFX Maestro 1.1 software.

## 5. Conclusions

Bioactive compounds present in plant extracts have been receiving significant attention lately. Plant metabolites can have a huge impact on the growth and metabolism of fungal pathogens, therefore being potential biocontrol agents in modern sustainable agriculture. Plant metabolites used in this study influenced the growth of the *Fusarium proliferatum* strain and fumonisin biosynthesis in vitro. Consequently, gene expression levels were affected. However, the effects observed were lower than those reported in previous studies analyzing pure extracts or their fractions. Therefore, it is likely that synergistic effects occur between tested compounds and other plant metabolites present in the original extracts.

## Figures and Tables

**Table 1:** Plant metabolites selected for the study and parameters used for identification of bioactive compounds.

Plant Metabolite	Molecular Formula	Ionization Mode	Parent Ion [*m*/*z*]	Product Ions [*m*/*z*]
Quercetin-3-glucoside	C_21_H_20_O_12_	[M-H]^-^	463.1	301.0
Kaempferol-3-rutinoside	C_27_H_30_O_15_	[M-H]^-^	593.1	285.0
Isorhamnetin-3-*O*-rutinoside	C_28_H_32_0_16_	[M-H]^-^	623.2	300.0
Ferulic acid	C_10_H_10_O_4_	[M-H]^-^	193.1	177.4
Chlorogenic acid	C_16_H_18_O_9_	[M-H]^-^	353.1	191.1
Neochlorogenic acid	C_16_H_18_O_9_	[M-H]^-^	353.1	191.1; 179.0
Protodioscin	C_51_H_84_O_22_	[M + H]^+^	1050.2	741.9
DIMBOA	C_9_H_9_NO_5_	[M-H]^-^	210.0	149.0

**Table 2:** Primer sequences, concentrations, and annealing temperatures used for RT-qPCR analyses in the present study.

Primer Name	Sequence 5′-3′	Concentration [nM]	Annealing Temperature [°C]
*FeSOD_F*	TGCCAGAGTTCTTGTCCCTG	300	64
*FeSOD_R*	ATGAGGACTTTGGCTCCTTC		
*SSC1_F*	AGGACGCAAGTTCAAGGATG	300	64
*SSC1_R*	AGGACGAAACCACCAATCTG		
*HSP88_F*	TTCAGCTGTGCCATTCTCTC	300	64
*HSP88_R*	ACGTTGCCCTTGTTGAAGAC		
*HSP70_F*	TGTCTTCGATGCCAAGTGA	300	64
*HSP70_R*	CAATCTTGAAGGGCCAAGAC		
*CPR6_F*	AATGAGGCTAACACCCTTGC	300	64
*CPR6_R*	TGTAGGCAGCCTTTTCCTTG		
*UOR_F*	CGATATTCGAGTCGAGGAGTG	300	66
*UOR_R*	CTATGATCTTCGAGAGGCTT		
*SpD_F*	GTCGCCATCATGTCTGACTATC	250	64
*SpD_R*	CAGCATCAACGAGTGTCTTG		
*FUM1_F*	TTCTCCCAGCCACTTGTATGC	500	62
*FUM1_R*	CCATCTCCCTTGTACTGTTGA		
*FUM6_F*	ATGACCGTTCGCCCAGTAGG	500	66.4
*FUM6_R*	CGTGTCCTTGATGAAGTCCCA		
*FUM8_F*	TCAACAAGCGAGCGACAACACA	500	68
*FUM8_R*	CCAGCCATACTTCCCATAAGTC		
*FUM19_F*	TCTCCCAACGCCCTGCC	500	62
*FUM19_R*	GGTGAGAAACTGACCGA		
*H3_F*	ACTAAGCAGACCGCCCGCAGG	250	62
*H3_R*	GCGGGCGAGCTGGATGTCTT		

## Data Availability

Not applicable.

## Supplementary Materials

The supporting information can be downloaded at: https://www.mdpi.com/article/10.3390/ijms24033002/s1.
