# Supplementary material for: Plant Metabolites Affect Fusarium proliferatum Metabolism and In Vitro Fumonisin Biosynthesis
Source: Int J Mol Sci. 2023 Feb 3;24(3):3002. doi: 10.3390/ijms24033002 (PMC9917803; doi:10.3390/ijms24033002)
Supplement: Supplementary file 1 [file ijms-24-03002-s001.zip › ijms-2072064-supplementary.pdf]

Supplementary materials

Plant metabolites affect *Fusarium proliferatum* metabolism and *in vitro* fumonisin biosynthesis

Natalia Witaszak [1†], Justyna Lalak-Kańczugowska [2†], Agnieszka Waśkiewicz [3], Jan Bocianowski [4] and Łukasz Stępień [1,*]

Table S1. Changes in the expression of primary metabolism-related genes in *F. proliferatum* KF 3360 strain after addition of plant bioactive compounds at 2, 24, 72 and 120 hours.

Metabolite	Normalized expression	S.D.	C_t	C_t S.D.	*p* value	
		FeSOD				
0h DIMBOA	0,95059	0,51435	30,4	0,71725	0,262228	
2h DIMBOA	0,77845	0,13376	29,8	0,17737	0,120437	
12h DIMBOA	6,89756	7,26233	30,28	1,31757	0,023092	*
24h DIMBOA	1,70369	0,40686	31,35	0,24473	0,402533	
3d DIMBOA	0,34272	0,32061	29,6	1,33104	0,007134	**
0h protodioscin	0,85316	0,5685	31,05	0,95939	0,387738	
2h protodioscin	0,49613	0,06289	31,5	0,16896	0,03707	*
12h protodioscin	6,03821	5,36608	30,1	1,16674	0,022039	*
24h protodioscin	1,8865	0,72508	30,45	0,40023	0,167819	
3d protodioscin	0,2334	0,07341	30,75	0,45438	0,000024	**
0h K-3-Rut	1,28032	2,01123	30,77	2,0258	0,385379	
2h K-3-Rut	0,2473	0,31764	32,15	1,85514	0,051541	
12h K-3-Rut	3,36582	1,40859	31,85	0,33509	0,080619	
24h K-3-Rut	0,60133	0,89288	32,57	2,1464	0,882337	
3d K-3-Rut	0,70263	0,99659	28,67	1,88488	0,716088	
0h Q-3-Glc	0,58683	0,54692	33,39	0,92853	0,201518	
2h Q-3-Glc	0,23138	0,20426	33,49	1,01457	0,004715	**
12h Q-3-Glc	1,29584	0,30004	33,37	0,29465	0,734099	
24h Q-3-Glc	0,68821	0,52311	33,22	0,96803	0,243465	
3d Q-3-Glc	0,21209	0,28056	32,08	1,89493	0,006373	
0h Iso-3-Rut	2,45012	3,63897	30,56	1,67186	0,137591	
2h Iso-3-Rut	0,64486	1,82404	30,17	3,44575	0,358268	
12h Iso-3-Rut	1,76947	2,51386	33,61	1,95212	0,243502	
24h Iso-3-Rut	1,22116	0,98269	32,5	1,13426	0,513925	
3d Iso-3-Rut	0,69497	0,45049	29,49	0,74643	0,329626	
0h FA	1,80638	1,24429	31,99	0,55528	0,591312	
2h FA	1,21874	0,74411	30,25	0,84459	0,503678	
12h FA	2,0275	1,03459	33,87	0,66154	0,646067	
24h FA	2,41565	4,02025	29,98	1,85839	0,073968	
3d FA	0,70192	0,61363	29,87	1,03486	0,703518	
0h ClA	9,18545	2,92143	28,21	0,06645	0,000015	**
2h ClA	0,80337	0,90701	31,2	1,60663	0,895404	
12h ClA	3,87283	2,02898	32,11	0,66563	0,050182	
24h ClA	6,22275	1,54384	28,6	0,21112	0,000003	**
3d ClA	1,10425	0,45048	30,01	0,47932	0,526457	

0h NClA	0,09718	0,07075	34,13	1,03203	0,035149	*
2h NClA	0,33404	0,18063	31,59	0,70426	0,01037	*
12h NClA	3,01709	1,86438	31,98	0,73017	0,080254	
24h NClA	1,30195	1,87678	30,81	1,66338	0,2724	
3d NClA	0,30732	0,16309	30,77	0,6248	0,000089	**
			CPR6			
0h DIMBOA	1,67338	1,10827	29,02	0,8692	0,180845	
2h DIMBOA	0,90673	0,14717	29,04	0,15125	0,007566	**
12h DIMBOA	2,00607	1,92441	30,79	1,11254	0,14142	
24h DIMBOA	0,57309	0,22754	31,48	0,49915	0,014358	*
3d DIMBOA	0,33481	0,08303	28,72	0,2556	0	**
0h protodioscin	1,02586	0,33195	30,18	0,44122	0,225967	
2h protodioscin	0,29551	0,2112	31,61	0,98989	0,004113	**
12h protodioscin	2,22727	1,95823	30,29	1,10585	0,19935	
24h protodioscin	0,57963	0,40909	30,75	0,90725	0,016277	*
3d protodioscin	0,51119	0,02928	28,7	0,07756	0,000085	**
0h K-3-Rut	2,00734	2,83793	29,53	1,69713	0,417229	
2h K-3-Rut	0,45595	0,69947	30,67	2,1283	0,973468	
12h K-3-Rut	2,41143	1,13549	31,05	0,43969	0,790496	
24h K-3-Rut	0,56929	0,41687	31,22	1,01584	0,208942	
3d K-3-Rut	0,66316	0,40591	27,87	0,34694	0,022646	
0h Q-3-Glc	1,41807	1,3226	31,45	0,89301	0,643356	
2h Q-3-Glc	0,15714	0,15802	33,35	1,18163	0,001227	**
12h Q-3-Glc	0,40532	0,3302	33,66	1,12071	0,241349	
24h Q-3-Glc	0,1313	0,12012	34,07	1,1678	0,015356	*
3d Q-3-Glc	0,16376	0,06799	31,43	0,51971	0,000004	**
0h Iso-3-Rut	2,97524	4,07916	29,67	1,41271	0,203787	
2h Iso-3-Rut	0,6676	1,54228	29,55	2,43705	0,529859	
12h Iso-3-Rut	0,72655	0,50686	33,47	0,75638	0,211148	
24h Iso-3-Rut	1,09605	0,56811	31,2	0,67654	0,910048	
3d Iso-3-Rut	0,71282	0,7553	28,56	1,36977	0,987843	
0h FA	3,36399	2,40993	30,44	0,61374	0,314346	
2h FA	1,62243	0,64294	29,26	0,49357	0,170024	
12h FA	0,93662	0,5057	33,55	0,68287	0,495543	
24h FA	2,04041	3,43089	28,88	1,83197	0,111272	
3d FA	0,39731	0,27653	29,75	0,67954	0,004707	**
0h ClA	2,88251	5,18759	29,31	2,46009	0,110002	
2h ClA	0,2951	0,32612	32	1,50903	0,078395	
12h ClA	0,38775	0,2234	34,03	0,72062	0,215118	
24h ClA	3,09628	0,6629	28,3	0,10396	0,005695	**
3d ClA	0,23931	0,18297	31,2	1,00884	0,000274	***
0h NClA	0,17341	0,17703	32,58	1,4033	0,090148	
2h NClA	0,16351	0,18058	31,97	1,49821	0,11656	
12h NClA	2,03415	1,94158	31,26	1,2295	0,170408	
24h NClA	0,48139	0,68889	30,84	1,57903	0,313744	
3d NClA	0,09374	0,05997	31,45	0,7786	0	**
			Hsp70			
0h DIMBOA	2,49203	0,81491	26	0,38951	0,152357	

2h DIMBOA	1,08879	0,2923	25,79	0,38081	0,22969	
12h DIMBOA	2,25	2,45493	27,38	1,51564	0,487005	
24h DIMBOA	0,71263	0,39853	28,58	0,84616	0,072086	
3d DIMBOA	0,39861	0,30073	26,24	1,16776	0,001188	**
0h protodioscin	2,53097	0,35803	26,52	0,20261	0,283588	
2h protodioscin	0,70823	0,14136	27,63	0,30699	0,000534	**
12h protodioscin	3,34623	2,48554	26,34	1,01995	0,232502	
24h protodioscin	1,10069	0,33198	27,07	0,22369	0,362961	
3d protodioscin	0,6755	0,13076	26,05	0,30649	0,002776	**
0h K-3-Rut	2,85797	3,6851	26,66	1,70761	0,083099	
2h K-3-Rut	0,67134	0,99059	27,32	2,33952	0,506013	
12h K-3-Rut	3,62876	1,29164	27,21	0,11518	0,010103	*
24h K-3-Rut	1,51459	0,09405	27,07	0,08704	0,141781	
3d K-3-Rut	0,5736	0,62899	25,78	1,49717	0,427415	
0h Q-3-Glc	0,96362	0,69453	30,02	0,3993	0,469194	
2h Q-3-Glc	0,22725	0,1931	30,41	1,0467	0,000004	**
12h Q-3-Glc	0,61751	0,13347	30,17	0,29531	0,233768	
24h Q-3-Glc	0,25133	0,11752	30,85	0,47376	0,001181	**
3d Q-3-Glc	0,51466	0,36626	27,79	1,09271	0,037366	*
0h Iso-3-Rut	2,57004	3,05723	27,64	1,17282	0,077418	
2h Iso-3-Rut	0,39749	0,84578	27,51	2,35704	0,587909	
12h Iso-3-Rut	0,41181	0,28871	31,57	0,86351	0,054623	
24h Iso-3-Rut	0,5652	0,2128	29,68	0,52543	0,116106	
3d Iso-3-Rut	0,75354	0,3068	26,22	0,17456	0,006186	**
0h FA	1,09073	2,05265	30,08	2,84498	0,965978	
2h FA	0,51836	0,70137	28,18	2,12843	0,058075	
12h FA	0,60951	0,43397	31,45	1,07141	0,036581	*
24h FA	1,62041	2,57198	26,33	1,87864	0,126012	
3d FA	0,86455	0,49694	26,44	0,44441	0,318515	
0h ClA	5,76824	1,61897	25,25	0,25858	0,000016	
2h ClA	2,09199	1,40581	25,08	0,88095	0,020494	*
12h ClA	3,79234	2,27119	26,32	0,83088	0,050483	
24h ClA	0,74387	0,55665	27,09	1,14891	0,471147	
3d ClA	0,93492	0,77284	26,44	1,16899	0,669546	
0h NClA	0,86903	1,39725	28,87	2,50191	0,524911	
2h NClA	0,31818	0,2666	29,34	1,29125	0,000709	**
12h NClA	0,58016	0,53054	30,62	1,39577	0,15585	
24h NClA	0,24937	0,40771	29,28	2,57469	0,250867	
3d NClA	0,84248	0,40147	27,36	0,65533	0,488589	
Hsp88						
0h DIMBOA	3,77246	1,18295	26,6	0,33747	0,059641	
2h DIMBOA	1,28673	0,26786	27,08	0,25225	0,839499	
12h DIMBOA	4,78818	5,10546	27,96	1,37607	0,169507	
24h DIMBOA	0,99488	0,56039	29,11	0,79811	0,42427	
3d DIMBOA	0,50639	0,32809	26,57	0,92987	0,013343	*
0h protodioscin	3,37116	0,64557	27,28	0,2696	0,274252	
2h protodioscin	0,88891	0,38538	28,71	0,63962	0,408927	
12h protodioscin	5,76696	3,79341	27,3	0,80528	0,080012	

24h protodioscin	1,33005	0,58075	27,91	0,51341	0,923908	
3d protodioscin	1,0055	0,04324	26,16	0,06102	0,001219	**
0h K-3-Rut	1,41221	3,25996	28,88	3,26143	0,177181	
2h K-3-Rut	0,40701	0,62275	29,51	2,27029	0,998523	
12h K-3-Rut	6,66464	2,44063	28,02	0,16719	0,011674	*
24h K-3-Rut	0,51283	0,55611	29,8	1,60961	0,311051	
3d K-3-Rut	0,80115	0,72423	26	1,05518	0,94816	
0h Q-3-Glc	0,37303	0,44472	32,39	1,45877	0,132523	
2h Q-3-Glc	0,08522	0,09832	33,11	1,51732	0,000164	**
12h Q-3-Glc	1,03903	0,35483	30,93	0,4801	0,337652	
24h Q-3-Glc	0,19168	0,12747	32,13	0,83042	0,004497	**
3d Q-3-Glc	0,35421	0,29409	28,94	1,20372	0,036819	*
0h Iso-3-Rut	4,62895	5,88719	27,87	1,28488	0,03559	*
2h Iso-3-Rut	0,58745	1,39898	28,35	2,71789	0,644356	
12h Iso-3-Rut	0,82115	0,6514	31,98	0,97815	0,174838	
24h Iso-3-Rut	1,30135	0,40367	29,39	0,37596	0,633587	
3d Iso-3-Rut	0,93532	0,44219	26,59	0,39245	0,809305	
0h FA	2,86127	4,71376	29,6	2,29416	0,093846	
2h FA	0,61443	0,99132	29,31	2,38115	0,906716	
12h FA	1,12069	0,54616	31,99	0,64121	0,085868	
24h FA	2,94745	4,83598	26,61	1,86315	0,062741	
3d FA	1,02068	0,71436	26,87	0,72424	0,706091	
0h ClA	1,29266	2,54181	29,31	2,88214	0,243887	
2h ClA	0,69326	0,51282	29,49	1,05768	0,656707	
12h ClA	0,80186	1,01627	31,64	1,84493	0,39137	
24h ClA	5,18418	1,12819	25,75	0,12538	0,000019	**
3d ClA	0,53685	0,48338	28,64	1,28955	0,378877	
0h NClA	0,17232	0,09151	31,63	0,7592	0,034303	*
2h NClA	0,21958	0,42276	30,29	2,83751	0,502226	
12h NClA	1,72088	2,51183	30	2,10186	0,538643	
24h NClA	1,17547	1,98071	27,91	2,14408	0,229107	
3d NClA	0,37789	0,28458	28,04	1,0204	0,012563	*
		SpD				
0h DIMBOA	0,96632	0,25139	30,16	0,20596	0,18807	
2h DIMBOA	1,41883	0,34528	28,07	0,30065	0,133132	
12h DIMBOA	2,36089	1,72266	30,34	0,71673	0,7076	
24h DIMBOA	1,38453	0,55735	30,92	0,51964	0,923513	
3d DIMBOA	0,34913	0,14022	28,47	0,51971	0,000001	**
0h protodioscin	2,14166	0,57936	29,52	0,37462	0,124834	
2h protodioscin	0,59833	0,2417	30,33	0,56989	0,045307	*
12h protodioscin	3,27389	2,48296	29,51	0,93914	0,668604	
24h protodioscin	1,73383	1,11757	29,86	0,83405	0,43211	
3d protodioscin	0,62048	0,09166	28,24	0,2092	0,002931	**
0h K-3-Rut	3,394	5,23885	26,21	2,10589	0,116052	
2h K-3-Rut	0,53542	0,79403	27,61	2,27713	0,874494	
12h K-3-Rut	2,61398	1,00934	27,5	0,25561	0,531774	
24h K-3-Rut	0,27914	0,34137	28,7	1,87874	0,034572	*
3d K-3-Rut	0,29208	0,25288	25,31	1,01626	0,000063	**

0h Q-3-Glc	1,456	1,10891	29,09	0,54089	0,941087	
2h Q-3-Glc	0,10427	0,08116	31,45	0,86785	0,000007	**
12h Q-3-Glc	0,19573	0,04306	31,63	0,29286	0,349678	
24h Q-3-Glc	0,13407	0,0552	30,72	0,3074	0,000742	**
3d Q-3-Glc	0,22298	0,17193	27,51	1,15231	0,000014	**
0h Iso-3-Rut	2,36209	3,92065	30,4	1,952	0,079002	
2h Iso-3-Rut	0,3865	1,21116	30,02	3,89206	0,601725	
12h Iso-3-Rut	1,17555	1,028	32,68	1,07244	0,380106	
24h Iso-3-Rut	2,32418	1,00186	30,83	0,55721	0,085532	
3d Iso-3-Rut	0,57133	0,28034	28,66	0,41949	0,001046	**
0h FA	3,74698	2,68931	30,71	0,61649	0,00492	**
2h FA	0,74927	1,03644	30,06	1,94973	0,634395	
12h FA	2,70448	2,22082	31,94	1,12207	0,302681	
24h FA	3,03814	4,80722	28,95	1,67325	0,120843	
3d FA	0,64689	0,46689	28,88	0,7371	0,090925	
0h ClA	1,57259	2,38823	30,54	2,11158	0,091758	
2h ClA	0,19453	0,10258	32,32	0,69372	0,001346	**
12h ClA	0,6255	0,34332	33,21	0,69445	0,384254	
24h ClA	2,16932	0,71917	29,41	0,37516	0,019106	*
3d ClA	0,16177	0,10706	31,63	0,87783	0	**
0h NClA	0,12745	0,06531	33,47	0,6992	0,00368	**
2h NClA	0,2996	0,70654	30,84	3,33505	0,514688	
12h NClA	3,66461	6,0577	30,22	2,29425	0,1324	
24h NClA	2,46141	3,18913	29,19	1,36645	0,130005	
3d NClA	0,32184	0,26195	29,58	1,07076	0,000544	**
			SSC1			
0h DIMBOA	2,60709	0,7635	25,77	0,3038	0,395598	
2h DIMBOA	1,01366	0,27869	25,89	0,37989	0,160023	
12h DIMBOA	1,92196	1,75971	27,4	1,15011	0,925937	
24h DIMBOA	0,30159	0,15719	28,88	0,75792	0,002343	**
3d DIMBOA	0,24201	0,09398	25,48	0,53941	0	**
0h protodioscin	2,31103	0,50339	26,48	0,32138	0,734175	
2h protodioscin	0,69713	0,07098	27,58	0,13708	0,016196	*
12h protodioscin	3,39326	2,59263	26,14	1,02532	0,357816	
24h protodioscin	0,62554	0,26642	26,96	0,51033	0,020722	*
3d protodioscin	0,40908	0,0273	25,3	0,10069	0,000082	**
0h K-3-Rut	1,99553	2,82144	29,92	1,73755	0,099512	
2h K-3-Rut	0,41897	0,49386	30,49	1,67326	0,03172	*
12h K-3-Rut	3,09094	1,32547	30,49	0,35521	0,568184	
24h K-3-Rut	0,54282	0,85226	31,97	2,23109	0,939223	
3d K-3-Rut	0,49818	0,56699	28,07	1,40923	0,245712	
0h Q-3-Glc	0,78328	0,97449	32,71	1,48499	0,877658	
2h Q-3-Glc	0,17568	0,13315	32,95	0,76203	0,002957	**
12h Q-3-Glc	0,49179	0,41798	33,24	1,19783	0,158185	
24h Q-3-Glc	0,54518	0,26162	32,79	0,45122	0,092775	
3d Q-3-Glc	0,2158	0,11914	30,91	0,74204	0,000001	**
0h Iso-3-Rut	5,90847	7,79994	26,14	1,43658	0,075055	
2h Iso-3-Rut	0,56618	1,23646	26,9	2,4044	0,439236	

12h Iso-3-Rut	0,69289	0,84576	30,42	1,7484	0,052638	
24h Iso-3-Rut	0,55478	0,19547	28,65	0,46617	0,122969	
3d Iso-3-Rut	0,38594	0,17038	25,73	0,31203	0,000003	**
0h FA	4,7343	2,8192	27,52	0,25536	0,018397	
2h FA	0,61937	0,13089	27,82	0,17603	0,031388	*
12h FA	0,58168	0,24006	31,18	0,53075	0,361389	
24h FA	1,17602	1,95022	25,87	1,96333	0,244431	
3d FA	0,38791	0,24855	26,15	0,61193	0,000041	**
0h ClA	0,91412	1,27002	28,53	2,07946	0,904362	
2h ClA	0,2647	0,27	29,5	1,53715	0,011727	*
12h ClA	0,41185	0,33757	30,83	1,19949	0,201906	
24h ClA	2,2585	0,70987	24,85	0,37186	0,003474	**
3d ClA	0,34408	0,30473	27,18	1,311	0,003461	**
0h NClA	0,08454	0,04405	31,07	0,68898	0,054133	
2h NClA	0,09187	0,19037	29,63	2,83174	0,003152	**
12h NClA	0,6123	1,02243	29,37	2,24381	0,513843	
24h NClA	0,46584	0,83087	27,06	2,14263	0,823169	
3d NClA	0,18795	0,17422	26,79	1,20249	0,000015	**
UOR						
0h DIMBOA	N/A	N/A	23,75	0,13742	0,913424	
2h DIMBOA	N/A	N/A	23,6	0,06986	0,920453	
12h DIMBOA	1,29956	0,54221	31,09	0,52868	0,751835	
24h DIMBOA	0,38465	0,06859	33,21	0,2391	0,004749	**
3d DIMBOA	0,60692	0,12205	29,04	0,28004	0,00476	**
0h protodioscin	N/A	N/A	24,24	0,04999	0,849568	
2h protodioscin	N/A	N/A	24,63	0,04031	0,907389	
12h protodioscin	1,73012	0,65596	30,29	0,51383	0,803382	
24h protodioscin	1,15704	0,27879	30,81	0,31799	0,808784	
3d protodioscin	0,61487	0,07078	29,62	0,16994	0,007977	**
0h K-3-Rut	1,91769	2,76116	29,27	1,85274	0,152386	
2h K-3-Rut	0,45773	0,63379	30,2	2,04647	0,696032	
12h K-3-Rut	3,78638	1,43034	30,06	0,21576	0,004069	**
24h K-3-Rut	1,17754	0,98627	31,27	1,23826	0,365511	
3d K-3-Rut	1,08634	1,46228	28,28	1,81154	0,260658	
0h Q-3-Glc	0,85945	0,92994	31,98	1,25052	0,774651	
2h Q-3-Glc	0,14799	0,13589	33,15	1,10323	0	**
12h Q-3-Glc	0,75514	0,24908	32,59	0,46005	0,348379	
24h Q-3-Glc	0,8549	0,60032	32,61	0,89201	0,655731	
3d Q-3-Glc	0,85585	0,22692	30,36	0,28866	0,213667	
0h Iso-3-Rut	0,91243	1,85787	31,12	2,67798	0,643539	
2h Iso-3-Rut	0,76688	1,9642	28,83	3,04576	0,218265	
12h Iso-3-Rut	1,35671	1,08939	32,42	0,99166	0,654737	
24h Iso-3-Rut	2,33725	0,53887	31,23	0,2146	0,003143	**
3d Iso-3-Rut	1,42559	0,70199	28,7	0,44086	0,052202	
0h FA	3,09074	1,97725	30,32	0,42431	0,017198	*
2h FA	0,75988	1,30222	29,87	2,52143	0,541595	
12h FA	1,61391	1,48162	32,63	1,31628	0,952267	
24h FA	2,55115	3,96102	29,53	1,68214	0,197298	

3d FA	1,83727	1,42162	28,73	0,87109	0,041766	*
0h ClA	0,74751	1,24767	30,96	2,42478	0,982482	
2h ClA	0,15401	0,06579	32,59	0,55866	0	**
12h ClA	N/A	N/A	N/A	N/A	N/A	
24h ClA	3,59091	1,04195	29,01	0,31018	0,000023	**
3d ClA	0,62075	0,368	31,15	0,80337	0,088712	
0h NClA	0,11045	0,06647	33,12	0,86464	0,128786	
2h NClA	0,09042	0,12519	32,47	2,01846	0	**
12h NClA	0,78157	1,26551	32,36	2,33661	0,863144	
24h NClA	1,1571	2,3086	30,64	2,65708	0,15667	
3d NClA	0,73315	0,61663	29,79	1,15774	0,771602	

Table S2. Changes in the expression of *FUM* genes in *F. proliferatum* KF 3360 strain after addition of plant bioactive compounds at day 3 and day 5.

Metabolite	Expression	Expression SD	Mean Cq	Cq SD	p-value
		FUM1 3d			
DIMBOA	1,02332	0,46404	27,24	0,15181	0,979348
Protodioscin	15,52234	15,6179	30,58	0,63208	0,001072
Q-3-Glc	0,38347	0,98018	32,25	1,14002	0,164051
K-3-Rut	1,06653	3,37778	27,54	4,403	0,177503
Iso-3-Rut	1,34449	1,73443	28,76	0,71227	0,313327
FA	1,3119	1,67873	27,96	0,8388	0,428815
ClA	1,62267	2,0587	30,88	1,67635	0,278763
NClA	1,18326	1,88155	30,29	0,06645	0,336627
		FUM6 3d			
DIMBOA	0,08404	0,0372	32,57	0,0529	0,043597
Protodioscin	6,85	9,3784	9,3784	33,98	1,48118
Q-3-Glc	0,74863	1,88979	33,02	0,98161	0,631141
K-3-Rut	21,58317	18,42456	24,93	0,16224	0,000144
Iso-3-Rut	1,42866	1,93379	30,4	0,9257	0,358642
FA	0,29562	0,33977	31,84	0,21196	0,028225
ClA	15,00953	8,47514	29,4	0,35143	0,001212
NClA	0,70342	1,24566	32,77	1,12637	0,641144
		FUM19 3d			
DIMBOA	4,83829	2,9261	27,63	0,59693	0,02496
Protodioscin	15,27055	20,10753	34,39	1,37884	0,122767
Q-3-Glc	2,40202	6,03695	32,24	0,92078	0,175832
K-3-Rut	2,06342	3,77489	29,22	2,33999	0,174384
Iso-3-Rut	7,11359	13,84535	28,99	2,21995	0,167067
FA	0,25422	0,3007	32,97	0,45559	0,07841
ClA	5,53208	3,82755	31,74	0,67547	0,042285
NClA	6,24952	18,59747	30,53	3,62949	0,186143
		FUM1 5d			
DIMBOA	0,05174	0,03352	33,62	0,01782	0,000441
Protodioscin	0,06603	0,05446	33,29	0,5989	0,006808

Q-3-Glc	0,75879	1,51765	29,71	2,58157	0,535325
K-3-Rut	N/A	N/A	N/A	N/A	N/A
Iso-3-Rut	0,23324	0,21982	32,36	0,77519	0,003889
FA	0,13744	0,13531	32,66	1,17394	0,007369
ClA	0,31382	0,19814	30,34	0,41879	0,000074
NClA	1,25799	2,18529	31,1	0,39357	0,340993
		FUM6 5d			
DIMBOA	0,0463	0,03042	34,3	0	N/A
Protodioscin	0,05553	0,02369	34,06	0,12664	0,00013
Q-3-Glc	0,29704	0,25323	31,58	1,10801	0,765363
K-3-Rut	0,22135	0,10401	33,3	0,40616	0,000567
Iso-3-Rut	0,11031	0,04624	0,04624	33,98	0,38957
FA	0,02895	0,01431	0,01431	35,43	0,63182
ClA	0,14459	0,07684	31,96	0,67318	0,000198
NClA	0,73741	0,61473	32,42	0,43107	0,641489
		FUM8 5d			
DIMBOA	0,2436	0,16004	33,65	0	N/A
Protodioscin	0,44133	0,39567	32,82	0,76493	0,349761
Q-3-Glc	0,85835	0,98678	31,8	1,02027	0,751796
K-3-Rut	2,67688	2,7755	31,46	0,87447	0,093227
Iso-3-Rut	0,42651	0,33859	33,78	0,16712	0,118758
FA	0,14569	0,18459	34,86	1,63813	0,063382
ClA	3,33136	2,08348	29,19	0,37536	0,009131
NClA	0,92721	1,61352	33,84	0	N/A

Table S3. Changes in fumonisin synthesis by *F. proliferatum* KF 3360 strain in liquid cultures after addition of plant bioactive compounds.

Compound	FBs [µg/ml]	0 h	2 h	12 h	24 h	3 d	5 d
DIMBOA	FB_1	0,19±0,4	0,13±0,0	0,14±0,0	0,16±0,0	0,61±0,2	5,22±1,5
	FB_2	0,03±0,1	0,02±0,0	0,02±0,0	0,02±0,0	0,04±0,0	0,48±0,1
	FB_3	0,01±0,0	0,01±0,0	0,01±0,0	0,01±0,0	0,02±0,0	0,27±0,0
Protodioscin	FB_1	0,27±0,3	0,35±0,4	0,42±0,4	0,55±0,5	6,24±6,1	2,86±2,9
	FB_2	0,04±0,0	0,06±0,1	0,07±0,1	0,06±0,1	0,34±0,3	0,49±0,5
	FB_3	0,02±0,0	0,03±0,0	0,03±0,0	0,03±0,0	0,12±0,1	0,12±0,1
Q-3-Glc	FB_1	0,02±0,0	0,02±0,0	0,01±0,0	0,01±0,0	0,11±0,1	2,90±2,8
	FB_2	n.d.	n.d.	n.d.	n.d.	0,01±0,0	0,31±0,0
	FB_3	n.d.	n.d.	n.d.	n.d.	n.d.	n.d.
K-3-Rut	FB_1	0,17±0,1	0,21±0,1	0,23±0,1	0,25±0,0	1,19±0,7	1,80±0,6
	FB_2	0,03±0,0	0,04±0,0	0,04±0,0	0,03±0,0	0,08±0,1	0,25±0,1
	FB_3	0,01±0,0	0,02±0,0	0,02±0,0	0,02±0,0	0,04±0,0	0,15±0,0
Iso-3-Rut	FB_1	0,16±0,2	0,18±0,3	0,02±0,3	0,21±0,3	2,09±1,9	4,69±1,7
	FB_2	0,02±0,0	0,02±0,0	0,03±0,0	0,03±0,0	0,16±0,1	0,57±0,3
	FB_3	0,01±0,0	0,01±0,0	0,01±0,0	0,01±0,0	0,06±0,0	0,25±0,1
FA	FB_1	0,02±0,0	0,02±0,0	0,02±0,0	0,02±0,0	0,82±1,0	3,01±2,8
	FB_2	n.d.	n.d.	n.d.	n.d.	0,07±0,0	0,34±0,2
	FB_3	n.d.	n.d.	n.d.	n.d.	0,03±0,0	0,20±0,2
ClA	FB_1	0,24±0,4	0,31±0,5	0,50±0,8	1,02±1,7	18,34±6,9	2,04±2,1
	FB_2	0,03±0,0	0,03±0,1	0,05±0,1	0,07±0,1	1,69±0,9	0,20±0,1

	FB_3	0,01±0,0	0,02±0,0	0,03±0,0	0,04±0,1	0,81±0,4	0,08±0,1
NClA	FB_1	0,03±0,0	0,02±0,0	0,03±0,0	0,03±0,0	0,06±0,0	4,21±3,2
	FB_2	n.d.	n.d.	n.d.	n.d.	0,01±0,0	0,57±0,3
	FB_3	n.d.	n.d.	n.d.	n.d.	n.d.	0,24±0,2
Control	FB_1	0,97±1,3	1,64±1,8	1,15±1,4	2,38±3,1	6,21±8,7	2,73±3,8
	FB_2	0,38±0,4	0,40±0,4	0,67±0,5	0,81±0,6	1,14±1,3	0,98±1,0
	FB_3	0,12±0,1	0,14±0,2	0,21±0,2	0,24±0,3	0,31±0,4	0,21±0,2
